# Identifying mood disorder subgroups at early risk of metabolic dysfunction: a cross-sectional cohort study in young people at early intervention services

**DOI:** 10.1136/bmjopen-2024-097140

**Published:** 2025-09-26

**Authors:** Sarah McKenna, Mirim Shin, Shin Ho Park, Alissa Nichles, Natalia Zmicerevska, Jacob Crouse, Connie Janiszewski, Minji Park, Elizabeth Phung, Frank Iorfino, Mathew Varidel, Elizabeth M Scott, Joanne Sarah Carpenter, Ian B Hickie

**Affiliations:** 1 Brain and Mind Centre, The University of Sydney, Camperdown, New South Wales, Australia

**Keywords:** Adolescents, Primary Health Care, PSYCHIATRY, Child & adolescent psychiatry, PUBLIC HEALTH

## Abstract

**Abstract:**

**Background:**

Severe mental disorders are associated with increased risk of metabolic dysfunction. Identifying those subgroups at higher risk may help to inform more effective early intervention. The objective of this study was to compare metabolic profiles across three proposed pathophysiological subtypes of common mood disorders (‘hyperarousal-anxious depression’, ‘circadian-bipolar spectrum’ and ‘neurodevelopmental-psychosis’).

**Methods:**

751 young people (aged 16–25 years; mean age 19.67±2.69) were recruited from early intervention mental health services between 2004 and 2024 and assigned to two mood disorder subgroups (hyperarousal-anxious depression (n=656) and circadian-bipolar spectrum (n=95)). We conducted cross-sectional assessments and between-group comparisons of metabolic and immune risk factors. Immune-metabolic markers included body mass index (BMI), fasting glucose (FG), fasting insulin, Homeostasis Model Assessment-Insulin Resistance (HOMA2-IR), C reactive protein and blood lipids.

**Results:**

Individuals in the circadian-bipolar spectrum subgroup had significantly elevated FG (*F*=5.75, p*=*0.04), HOMA2-IR (*F*=4.86, p=0.03) and triglycerides (*F*=4.98, p=0.03) as compared with those in the hyperarousal-anxious depression subgroup. As the larger hyperarousal-anxious depression subgroup is the most generic type, and weight gain is also a characteristic of the circadian-bipolar subgroup, we then differentiated those with the hyperarousal-anxious subtype on the basis of low versus high BMI (<25 kg/m^2^ vs ≥25 kg/m^2^, respectively). The ‘circadian-bipolar’ group had higher FG, FI and HOMA2-IR than those in the hyperarousal-anxious-depression group with low BMI.

**Conclusions:**

Circadian disturbance may be driving increased rates of metabolic dysfunction among youth with emerging mood disorders, while increased BMI also remains a key determinant. Implications for assessment and early interventions are discussed.

**STRENGTHS AND LIMITATIONS OF THIS STUDY**

- This study explores metabolic blood markers including insulin sensitivity (Homeostasis Model Assessment-Insulin Resistance (HOMA2-IR) and fasting insulin) in a youth population with major mood disorders.
- Study findings indicate that HOMA2-IR and fasting insulin are elevated in a group with bipolar and atypical depression symptoms as compared with a group with more common anxious-depression symptoms, whereas body mass index was not different, suggesting HOMA2-IR is a more sensitive early marker of dysfunction.
- Limitations include small group sizes of circadian-bipolar spectrum limiting the power of our analyses to detect group differences.
- Data were collected over 20 years and additional metabolic variables were only available for a subset (<25%) of participants.

## Introduction

 Metabolic syndrome and premature cardiovascular disease (pCVD) are highly prevalent among individuals with major mood disorders, particularly among those with bipolar and atypical mood disorders, and these rates have not improved over the past decade.^1 2^ It is unknown whether these problems co-occur and are driven by similar mechanisms or if they are causally related; thus, detailed monitoring from early stages of dysfunction is needed.^3^,^5^ Associations between mood disorders, cardiovascular risk and metabolic poor health are already present in child and youth populations, indicating that physical health comorbidities require early intervention.^2 6 7^ Moreover, rates of metabolic dysfunction are likely to be underestimated in youth with mood disorders as they are significantly less likely to receive cardiometabolic monitoring than those with psychotic disorders, even those on psychotropic medications.^8^,^10^ Identifying early markers of physical health comorbidities, particularly for illness subtypes with heightened risk, will enable detection, early intervention and prevention of serious health complications.

Current cardiometabolic guidelines for early intervention services emphasise monitoring of ‘downstream’ markers of metabolic dysfunction, such as body mass index (BMI), hip/waist circumference and fasting glucose, rather than early markers such as insulin resistance.^8 9^ However, young people in inpatient settings with more severe mental illnesses have significantly elevated insulin resistance (measured by Homeostasis Model Assessment-Insulin Resistance (HOMA2-IR), a measure of insulin-glucose homeostasis based on fasting insulin (FI) and glucose (FG)) as compared with those in ambulatory care despite not having raised BMI nor raised fasting glucose.^10^,^12^ Only 22% of the variance in HOMA2-IR was accounted for by BMI.^10^ Thus, although current guidelines emphasise monitoring of BMI and fasting glucose, these may only become elevated later in the course of illness after metabolic dysfunction has persisted for a prolonged period.^13^,^16^ Increased monitoring of sensitive early markers of metabolic dysfunction (such as HOMA2-IR) could drastically improve early detection and prevention of physical health comorbidities in youth with major mood disorders.^2 10^

Another problem is that illness subtypes associated with a heightened risk of comorbid health issues, such as bipolar and atypical mood disorders, may not be identified until adulthood. Early phases of major mood disorders are not easily distinguishable in youth populations, and identifying more serious illness trajectories is often unreliable until later stages of illness.^17 18^ Young people also undergo normative developmental changes, such as changes in sleep, mood and functioning, that may further complicate their presentation.^19^

Accordingly, we have previously outlined a transdiagnostic framework for major mood or psychotic disorders that includes three developmental pathways to early-onset major mood disorders based on typically observed illness trajectories and proposed pathophysiological mechanisms.^20^,^23^ A ‘hyperarousal-anxious depression’ subtype, the most common pathway, is characterised by child and adolescent anxiety, with the later development of depressive symptoms in early and mid-adolescence.^21^ Another is a ‘neurodevelopmental-psychosis’ subgroup that is related to childhood and adolescent onset related conduct behavioural and attention disorders and is later characterised by psychotic features and cognitive impairment. A third, ‘circadian-bipolar’ subgroup has been reviewed in the most detail by our group and involves mood disorders that are characterised by significant manic-like or atypical features.^24 25^ This ‘circadian-bipolar’ subgroup involves significant changes in energy or activity that are likely due to underlying perturbations of sleep-wake cycles and circadian rhythms.^24 25^ We previously reported no significant differences in BMI between the three illness groups.^26^ However, given that HOMA2-IR appears to be a more sensitive marker of metabolic dysfunction, it may also be better able to detect early differences between illness groups and identify those most in need of early monitoring and prevention strategies.

Taken together, the objectives of the current study were to compare metabolic risk factors between three proposed pathophysiological pathways to major mood disorders. We included common measures of metabolic health such as BMI and fasting glucose, alongside other metabolic blood markers, particularly HOMA2-IR, to explore whether this measure was a more sensitive indicator of emerging physical health comorbidities. There are higher rates of metabolic syndrome and pCVD among adults with bipolar and atypical mood disorders. Additionally, circadian rhythm disruption, for example, in shift workers, is linked to metabolic dysfunction.^27 28^ Thus, we hypothesised that individuals belonging to the circadian-bipolar spectrum illness group would have elevated metabolic risk factors, particularly elevated HOMA2-IR, as compared with other illness groups.

## Methods

Ethical approval for the study was obtained from the Human Research Ethics Committee of the Sydney Local Health District (2020/ETH01272). Informed consent was secured from all participants. A subset of participants from the Neurobiology Youth Follow-Up Study also consented to share their historical blood data from a prior study approved by the Human Research Ethics Committee of the University of Sydney (2012/1631). The current paper is reported according to Strengthening the Reporting of Observational Studies in Epidemiology guidelines (see online supplemental materials).^29^

### Study design

This cross-sectional study explored whether young people belonging to a circadian-bipolar spectrum illness group had poorer immune-metabolic measures as compared with a hyperarousal-anxious depression group and a neurodevelopmental-psychosis group. We also explored the extent to which HOMA2-IR can be considered a more sensitive early marker of metabolic dysfunction.

### Setting and participants

Study participants were drawn from a larger cohort of young people presenting to one of the youth mental health clinics associated with the Brain and Mind Centre^30 31^; headspace Camperdown (an early intervention service); Mind Plasticity (a private multidisciplinary clinic) and St Vincent’s private hospital (inpatient clinic). These clinics attract young people with subthreshold and full-threshold anxiety, mood or psychotic disorders. This cohort includes young people presenting to one of these services for mental healthcare, who underwent blood assessments between January 2004 and January 2024. Individuals were included in the current study if: (a) they had undergone a clinical assessment (and been assigned to a pathophysiological group) within 6 months of undergoing a blood test to assess metabolic and immune markers and (b) if they were aged under 25 years at the time of the blood test.

### Pathophysiological illness group

Participants were assigned to one of three illness groups based on clinical presentation: hyperarousal-anxious depression, circadian-bipolar spectrum or neurodevelopmental-psychosis. Allocation of participants was conducted by a trained clinical research staff who had received specific training and who was familiar with the pathophysiological mechanisms model as described in previous publications.^20^,^22^ Assignments are based on a description of the individuals’ entire clinical history from various data sources including clinician notes, structured clinical interviews and self-report data, taking into account normative developmental changes. Any cases with significant manic-like symptoms (manic, hypomanic or brief hypomanic phenomena) or significant atypical features (consistent with existing diagnostic criteria^32 33^; eg, reduced activation, low energy or prolonged fatigue not restored by sleep, increased appetite or weight gain) are allocated to the ‘circadian-bipolar spectrum’ subtype. Cases with a primary psychotic disorder or significant and persistent developmental difficulties (such as autism spectrum disorder, specific learning disability or low IQ) are allocated to the ‘neurodevelopmental psychosis’ subtype. Given the low prevalence of mania-like and psychotic-like symptoms within the mood disorder population, the hyperarousal-anxious depression group is the default option for those who have common anxiety and depressive symptoms but do not clearly have any of the defining features of the other two subtypes.

### Metabolic, immune and other hormonal measures

Blood samples were collected in a fasting state at a pathology laboratory. All participants in this study provided plasma FG and fasting insulin (FI). A subset of participants from the Neurobiology Youth Follow-up Study also provided cholesterol, triglycerides and systemic inflammation (C reactive protein (CRP)). HOMA2-IR was calculated using fasting blood glucose and insulin with the HOMA2 software V.2.2.3 (Hill *et al*). We defined ‘elevated risk’ by HOMA2-IR >1.5 and ‘high risk’ by HOMA2-IR >2, as outlined in our previous report.^13^ Low-grade inflammation was defined using CRP greater than 3 mg/L. Although we have noted that BMI may not be a sensitive marker of metabolic health, it remains the most commonly used measure of metabolic health in early intervention settings, so was also collected. Height and weight measures were collected by direct measurement or self-report for calculation of BMI (weight alone was not included in our analyses) using the formula: weight (kg) ÷ height (m^2^).

### Statistical analysis

A power analysis was performed using G*Power^34^ to determine the sample size necessary to detect a medium effect size (*F*=0.200) for an analysis of covariance (ANCOVA) comparing three illness groups while adjusting for two covariates (age and gender). The analysis assumed a significance level of α=0.05 and a desired power of 0.80. Based on these parameters, the required sample size was calculated to be 304 participants, suggesting each group required around 100 participants to allow sufficient power to detect group differences.

Statistical analyses were performed using SPSS V.29.^35^ Continuous measures are reported as medians with IQRs or mean± SD, while categorical measures are reported as N (%). We used partial correlation coefficients to conduct pairwise comparisons between immune-metabolic markers while controlling for age and gender. To explore differences between illness groups, we conducted ANCOVA controlling for age and gender.

Several variables had a large proportion of missing data (see table 1; BMI=46.1% missing; CRP=65.4% missing; triglycerides=70.4% missing; high-density lipoprotein (HDL) and low-density lipoprotein (LDL) cholesterol=75% missing; total cholesterol=70.4% missing). We used pairwise deletion to handle missing data to preserve data as compared with listwise deletion.

**Table 1:** Descriptive comparison of demographics and immune-metabolic markers between each illness pathway

	% Missing	Full sample (n=751)	Hyperarousal-anxious depression (n=656)	Circadian-bipolar spectrum (n=95)
		M (SD)	M (SD)	M (SD)
Age	0.00%	19.67 (2.69)	19.49 (2.67)	20.92 (2.54)
12–18 (N(%))		309 (41.15%)	338 (89.42%)	26 (6.88%)
18–24 (N(%))		442 (58.85%)	318 (77.18%)	69 (16.75%)
Female (N(%))	0.10%	517 (68.84%)	446 (67.98)	71 (74.70)
BMI	47.67%	23.78 (33.33%)	23.54 (4.84)	25.41 (6.75)
n≥25 (%)		131 (17.44%)	109 (16.62)	22 (23.16)
Fasting glucose	0.00%	4.56 (0.49)	4.54 (0.48)	4.67 (0.55)
Fasting insulin	0.00%	9.45 (5.25)	9.34 (5.21)	10.19 (5.49)
HOMA2-IR	0.00%	1.19 (0.66)	1.18 (0.65)	1.29 (0.70)
n≥2 (%)		77 (10.25%)	64 (9.76)	13 (13.68)
n≥1.5 (%)		179 (23.83%)	148 (22.56)	29 (30.53)
CRP	66.18%	2.45 (3.16)	2.41 (3.12)	2.74 (3.48)
n≥3 (%)		64 (24.24%)	55 (8.38)	9 (9.47)
Triglycerides	71.90%	1.13 (0.73)	1.07 (0.72)	1.29 (0.76)
HDL cholesterol	76.03%	1.43 (0.36)	1.43 (0.34)	1.46 (0.43)
LDL cholesterol	76.03%	2.66 (0.72)	2.64 (0.74)	2.73 (0.66)
Total cholesterol	71.90%	4.57 (0.91)	4.50 (0.94)	4.76 (0.80)

BMI, body mass index; CRP, C reactive protein; HDL, High-density lipoprotein ; HOMA2-IR, Homeostasis Model Assessment-Insulin Resistance; LDL, Low-density lipoprotein.

## Results

The eligible sample included a total of 790 adolescents and young adults aged 16–25 years. Of these, 656 (83.0%; 67.98% female; M_age_=19.49 ± 2.67) were assigned to the hyperarousal-anxious depression group, 95 (12.0%; 74.70% female; M_age_=20.92 ± 2.54) were assigned to the circadian-bipolar spectrum group and 39 (4.9%; 38.50% female; M_age_=20.85 ± 2.67) were assigned to the neurodevelopmental-psychosis group (trends in group assignments across time are summarised online supplemental table S1 an figure S1. Given the small group size, the neurodevelopmental-psychosis illness group was underpowered and was not included in analyses. As such, 751 individuals were included in the current study.

### Pairwise comparisons between immune and metabolic markers

**Figure 1:**
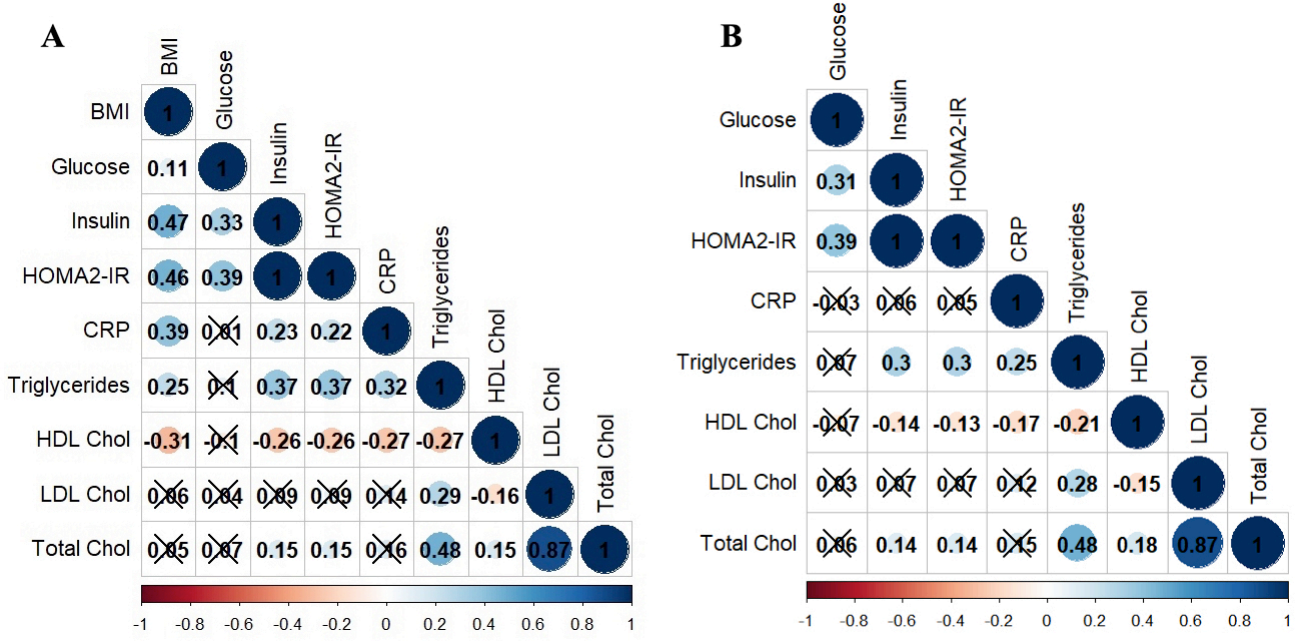
Partial correlations between immune-metabolic markers controlled for age and gender (**A**) and controlled for age, gender and BMI (B; crosses show non-significant relationships p>0.05). BMI, body mass index; CRP, C reactive protein; HOMA2-IR, Homeostasis Model Assessment-Insulin Resistance.

We first conducted pairwise comparisons, using partial correlation coefficients (controlling for age and gender) to examine relationships between BMI and immune and metabolic variables. As shown in panel A figure 1 (and in online supplemental table S2), BMI was significantly associated with immune-metabolic markers FG (*r*=0.107, p*=*0.012), FI (*r*=0.472, p*<*0.001), HOMA2-IR (*r*=0.465, p*<*0.001), CRP (*r*=0.392, p*<*0.001), triglycerides (*r*=0.246, p*<*0.001) and HDL cholesterol (*r*=−0.310, p*<*0.001).

We then explored the relative usefulness of FI and HOMA2-IR to FG as indicators of metabolic risk before and after controlling for BMI. FI was significantly correlated with FG (*r*=0.325, p*<*0.001), CRP (*r*=0.232, p*<*0.001), triglycerides (*r*=0.373, p<0.001), HDL cholesterol (*r*=−0.261, p*<*0.001) and total cholesterol (*r*=0.147, p=0.005). HOMA2-IR was also correlated with FG (*r*=0.390, p*<*0.001), CRP (*r*=0.223, p*<*0.001), HDL cholesterol (*r*=−0.257, p*<*0.001) and total cholesterol (*r*=0.150, p*=*0.004). FG was not associated with CRP or blood lipids.

Once BMI (panel B figure A; online supplemental table S3) was controlled for, FG was not associated with CRP or lipids and FI and HOMA2-IR were no longer associated with CRP. However, FI remained associated with triglycerides (*r=*0.373, p*<*0.001), HDL cholesterol (*r=*−0.134, p*=*0.025) and total cholesterol (*r*=0.143, p*=*0.012). While HOMA2-IR was associated with triglycerides (*r=*0.249, p*=*0.002), HDL cholesterol (*r=*−0.134, p*=*0.025) and total cholesterol (*r=*0.143, p*=*0.012). Taken together, this suggests that FG is an insensitive measure of metabolic dysfunction and that associations between elevated FI and HOMA2-IR and abnormal lipid profiles are relatively independent of BMI.

Once BMI (panel B Figure A; online supplemental table S3) was controlled for, FG was not associated with CRP or lipids and FI and HOMA2-IR were no longer associated with CRP. However, FI remained associated with triglycerides (*r=*0.37, p*<*0.001), HDL cholesterol (*r=*−0.13, p*=*0.03) and total cholesterol (*r*=0.14, p*=*0.01). While HOMA2-IR was associated with triglycerides (*r=*0.25, p*=*0.002), HDL cholesterol (*r=*−0.13, p*=*0.03) and total cholesterol (*r=*0.14, p*=*0.01). Taken together, this suggests that FG is an insensitive measure of metabolic dysfunction and that associations between elevated FI and HOMA2-IR and abnormal lipid profiles are relatively independent of BMI.

### Comparing demographic characteristics and metabolic and immune markers between illness groups (hyperarousal-anxious depression, neurodevelopmental-psychosis, circadian-bipolar spectrum)

The circadian-bipolar spectrum group was significantly older than the hyperarousal-anxious depression group (F=27.12, p<0.001). There was no difference in terms of gender (F=1.94, p=0.16).

**Figure 2:**
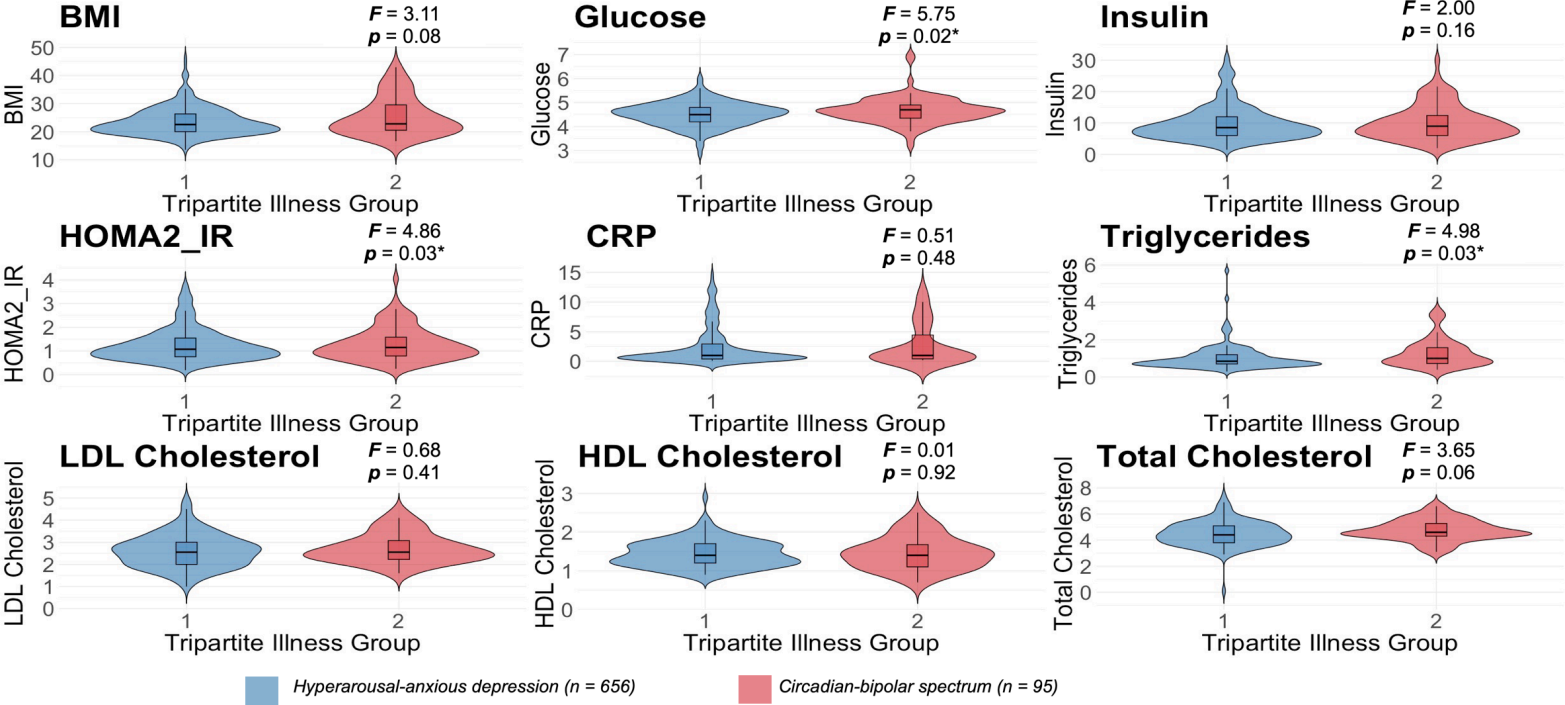
Comparisons of immune-metabolic markers between each illness group using analysis of cAnalysis of Covariance (controlled for age and sex). Note. *p<0.05; **p<0.01; ***p<0.001. BMI, body mass index; CRP, C reactive protein; HOMA2-IR, Homeostasis Model Assessment-Insulin Resistance.

We next explored whether immune and metabolic blood markers differed significantly between the illness groups after controlling for age and gender. As shown in figure 2, we found that FG (F=5.75, p=0.04), HOMA2-IR (F=4.86, p=0.03) and triglycerides (F=4.98, p=0.03) were significantly higher in the circadian-bipolar spectrum group as compared with the hyperarousal-anxious depression group. As shown in table 1, 30.53% of those in the circadian-bipolar spectrum group had HOMA2-IR>1.5 and 13.68% had HOMA2-IR>2 as compared with 22.56% and 9.76%, respectively, of those in the hyperarousal-anxious depression group.

### Subgroup analysis of hyperarousal anxious-depression individuals with low and high BMI as compared with circadian-bipolar spectrum and neurodevelopmental-psychosis groups

**Figure 3:**
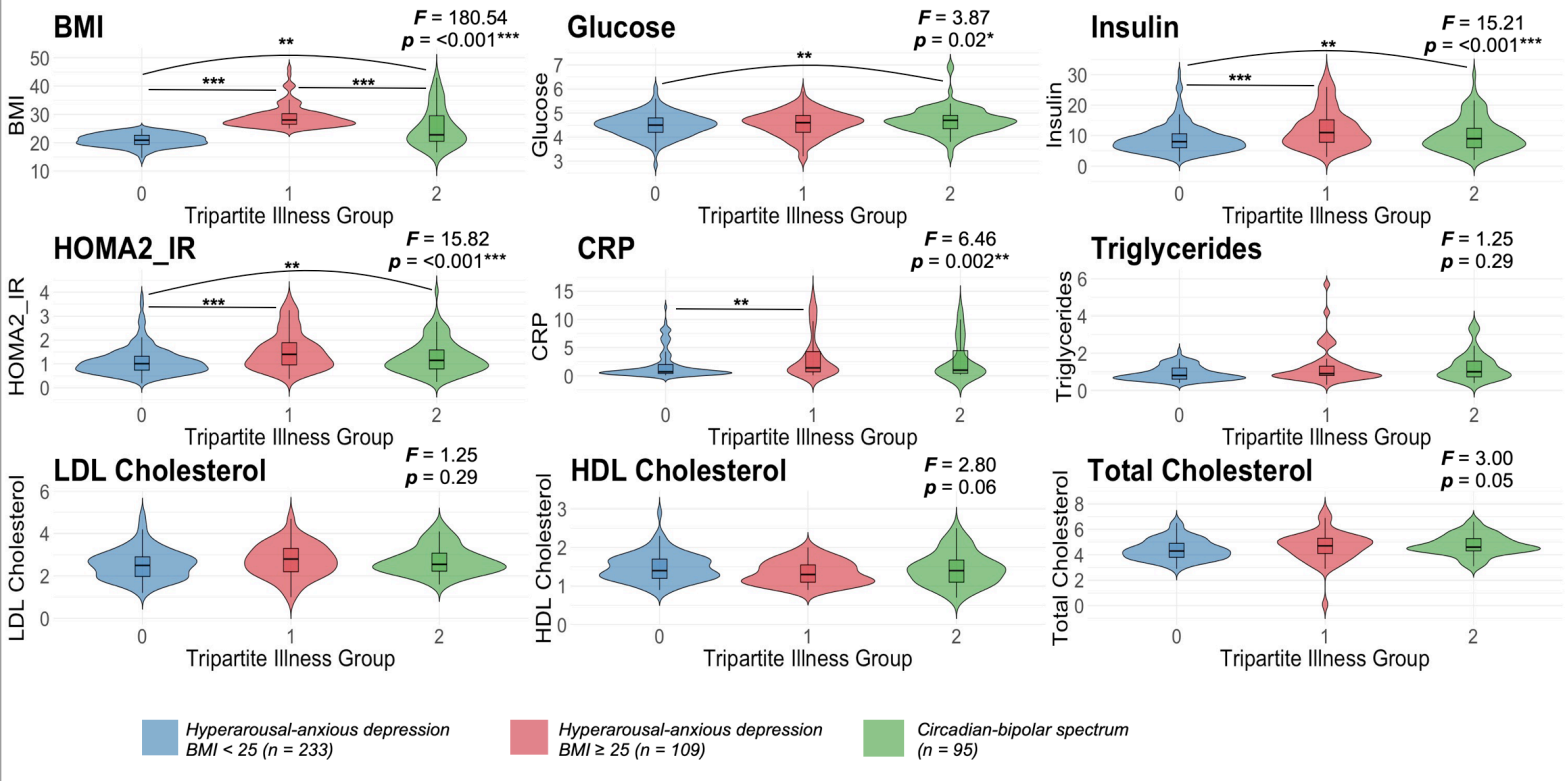
Comparisons of immune-metabolic markers between each illness group (including subgroup analysis of Hyperarousal-Anxious Depression with low (<25 kg/m^2^) and high (>25 kg/m^2^) BMI) using ANCOVA (controlled for age and sex) and post-hoc pairwise comparisons (Bonferroni corrected). Note. *p<0.05; **p<0.01; ***p<0.001. BMI, body mass index; CRP, C reactive protein; HOMA2-IR, Homeostasis Model Assessment-Insulin Resistance.

To further understand these differences, we explored immune-metabolic profiles of those in the hyperarousal anxious-depression group with low (BMI<25 kg/m^2^) versus those with high BMI (BMI>25 kg/m^2^). Age (F=16.19, p*<*0.001) significantly differed between the three groups as the circadian-bipolar spectrum group was older than the hyperarousal-anxious depression group with low (p<0.001) and high (p<0.01) BMI. Gender was also different between groups (*F*=6.67, p<0.01) as there were a higher proportion of males in the hyperarousal-anxious depression with high BMI as compared with the low BMI (p<0.01) and the circadian-bipolar spectrum (p<0.01) group. Previous data have shown that many individuals in this group are at an early stage of illness but develop more severe symptoms, such as more atypical depressive (notably including weight gain), manic or psychotic-like symptoms over time. Thus, individuals in this group may already be at heightened metabolic risk due to illness mechanisms, including emerging circadian disturbance. Figure 3 shows significant group differences were found for FG (*F*=3.87, p=0.02), FI (*F*=15.21, p<0.001), HOMA2-IR (*F*=15.82, p<0.001) and CRP (*F*=6.46, p=0.002) after controlling for age and sex. Posthoc pairwise comparisons showed that compared with the hyperarousal anxious-depression low BMI group, the high BMI group had elevated FI (p<0.001), HOMA2-IR (p<0.001) and CRP (p<0.01; see online supplemental table S4). Likewise, the circadian-bipolar spectrum group showed elevated FG (p=0.02), FI (p<0.01) and HOMA2-IR (p<0.01) as compared with the hyperarousal-anxious depression low BMI group.

## Discussion

The current study examined differences in metabolic blood markers between two proposed pathophysiological mechanism groups in a youth sample with emerging mood disorders. A key finding of our research was that insulin resistance (indexed by HOMA2-IR) was significantly heightened in a circadian-bipolar spectrum subgroup as compared with the more common hyperarousal anxious-depression group, whereas BMI was similar between groups. This is consistent with existing findings from our research and in other youth cohorts that mood symptoms are associated with poor metabolic health independently of adiposity in young people.^13 14 36 37^ Going forward, there are several important clinical and research advantages to monitoring insulin resistance in youth cohorts. In clinical settings, monitoring insulin resistance may lead to earlier identification of health problems and facilitate more effective prevention and early intervention approaches. More detailed longitudinal tracking is also required in research settings.^38^,^40^ Young people at early stages of major mood disorders have limited exposure to medication, smoking and other illness-related risk factors, making it easier to identify and track underlying mechanisms that are causally involved in linking mood disorders and metabolic dysfunction.

Another key finding of this study is that young people with circadian-bipolar type illness have heightened risk of metabolic dysfunction as compared with those with hyperarousal-anxious depression. This is consistent with findings from adult populations that rates of metabolic syndrome and pCVD are highest among those with bipolar and atypical mood disorders.^1 2^ Given that this study was cross-sectional, we cannot draw conclusions about which mechanisms are driving the observed differences between the groups. However, ongoing research is focused on the extent to which circadian rhythm disruption is driving ‘circadian-bipolar spectrum’ type mood disorders.^24 25^ Atypical and bipolar mood disorders are characterised by core clinical features, including hypersomnia, daytime fatigue, non-restorative sleep, reduced motor activity, somatic symptoms and appetite and weight change that are associated with circadian rhythm disruptions.^24 25^ Relationships between disrupted circadian systems and metabolic disruption (including insulin resistance) have also been widely reported.^41 42^ Detailed monitoring and longitudinal research in youth cohorts, tracking trajectories in mood disorder progression and comorbid physical health problems, will lead to improved understanding of potential mediating or moderating mechanisms underlying this relationship.

Notably, the observed differences between the circadian-bipolar spectrum illness group and the hyperarousal-anxious depression group were largely restricted to those individuals with low BMI. For all subtypes, there is a clear association between current weight and poor metabolic outcomes. However, we hypothesise that there may be individuals early in their course of illness and classified here within the hyperarousal-anxious depression group, who have not yet progressed to diagnostically clear atypical or mania-like symptoms required to be classed as having underlying circadian disturbance.^13 36^ Consistent with this interpretation, we have previously shown that 10% of those in the hyperarousal-anxious-depression group do transition to the circadian-bipolar spectrum group.^20^ In the current study, the prevalence of circadian disturbance in the hyperarousal-anxious-depression group (with or without high BMI) is not known. This is an avenue for future exploration. If our prediction is correct, this calls for more personalised assessment, prevention and early intervention of metabolic risk factors in youth presenting to early intervention services with circadian disturbance, irrespective of BMI and even before they meet full-threshold criteria for circadian-bipolar type illnesses.

This study had some important limitations. As it was cross-sectional, we are not able to explore longitudinal metabolic trajectories for individuals in different illness groups. It will be important to map metabolic trajectories to other variables to better understand drivers and correlates of deteriorations in metabolic health (eg, physical inactivity, genetic risk, objective circadian disturbance, medication exposures). Important confounders, most notably medication use or concurrent medical conditions and sociodemographic variables, such as ethnicity, were not accounted for in the analysis. Our statistical analyses were also affected by limitations of the data. For example, there were significantly more young people in the hyperarousal-anxious depression group (n=656) as compared with the neurodevelopmental-psychosis (n=39) and circadian-bipolar spectrum (n=95) groups. Based on our power analyses, the neurodevelopmental-psychosis group was underpowered to detect a moderate effect and we were not able to include them in our analyses. While this may reflect selection bias (eg, help-seeking behaviours, access to services, ambulatory and non-urgent care based) or potentially our assignment process (for which the hyperarousal-anxious depression group may be the ‘residual category’ for some cases that do not fit the other two groups). Additionally, several variables had a large proportion of missing data (BMI=46.1% missing; CRP=65.4% missing; triglycerides=70.4% missing; HDL and LDL cholesterol=75% missing; total cholesterol=70.4% missing), whereas FI, FG and HOMA2-IR had no missing data. Thus, certain analyses had greatly increased statistical power as compared with others. Given these limitations, further longitudinal research with youth cohorts is urgently needed to define illness subgroups most at risk of metabolic dysfunction and to identify potential causal mechanisms of this dysfunction.

Altogether, this cross-sectional cohort study provides novel evidence that metabolic risk factors vary between young people based on three proposed groups to major mood disorders. Those with circadian-bipolar type illnesses appear to be at highest risk of metabolic dysfunction. Additionally, for those with atypical, manic or psychotic symptoms, metabolic dysfunction is also linked to markers of inflammation. The current research was not able to identify potential mechanisms that could be driving this heightened risk but does suggest avenues to be explored in future longitudinal research. Our results also provide impetus for improved assessment and early intervention approaches that can lead to more personalised interventions and reduce the risk of pCVD associated with anxiety and major mood disorders.

## Supplementary material

10.1136/bmjopen-2024-097140 online supplemental file 1

10.1136/bmjopen-2024-097140 online supplemental file 2

## Data Availability

Data are available upon reasonable request.
