# Supplementary material for: Identifying mood disorder subgroups at early risk of metabolic dysfunction: a cross-sectional cohort study in young people at early intervention services
Source: BMJ Open. 2025 Sep 26;15(9):e097140. doi: 10.1136/bmjopen-2024-097140 (PMC12481334; doi:10.1136/bmjopen-2024-097140)
Supplement: online supplemental file 2 [file bmjopen-15-9-s002.docx]

**Table S1**

Proportion of individuals assigned to each illness group per year

| **Year** | **Neurodevelopmental-Psychosis** | **Anxiety-Depression** | **Circadian-Bipolar** |
| --- | --- | --- | --- |
| 2005 | 1.00 | 0.00 | 0.00 |
| 2006 | 0.00 | 1.00 | 0.00 |
| 2007 | 0.00 | 1.00 | 0.00 |
| 2008 | 0.00 | 0.67 | 0.33 |
| 2009 | 0.25 | 0.75 | 0.00 |
| 2010 | 0.14 | 0.43 | 0.43 |
| 2011 | 0.07 | 0.63 | 0.30 |
| 2012 | 0.06 | 0.65 | 0.28 |
| 2013 | 0.06 | 0.80 | 0.14 |
| 2014 | 0.03 | 0.88 | 0.09 |
| 2015 | 0.03 | 0.92 | 0.06 |
| 2016 | 0.08 | 0.81 | 0.11 |
| 2017 | 0.02 | 0.92 | 0.07 |
| 2018 | 0.00 | 0.96 | 0.04 |
| 2021 | 0.00 | 1.00 | 0.00 |
| 2022 | 0.20 | 0.71 | 0.09 |
| 2023 | 0.11 | 0.69 | 0.19 |
| 2024 | 0.07 | 0.78 | 0.15 |


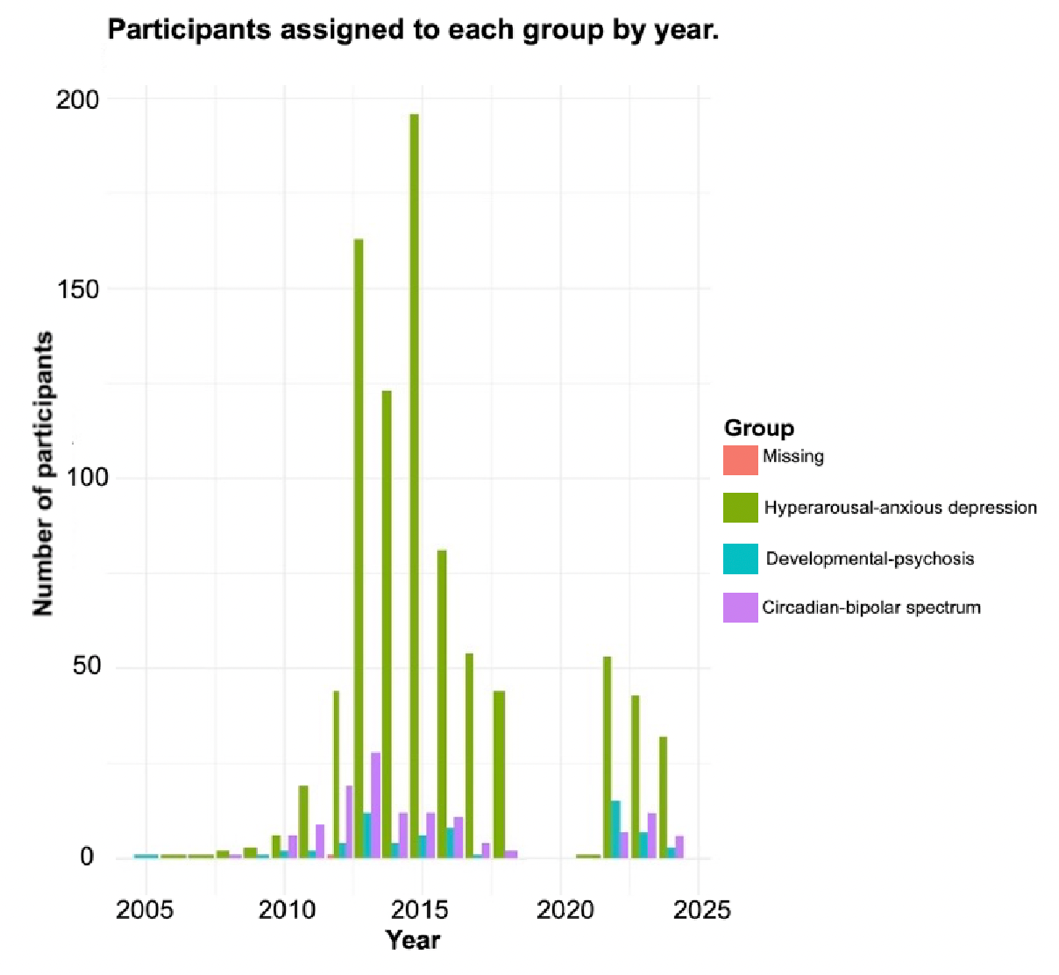


**Figure S1.** Distribution of participants assigned to each illness group per year.

| **Table S2**  Partial correlations between immune-metabolic markers controlled for age and gender (*r*(*p*)). | | | | | | | | |
| --- | --- | --- | --- | --- | --- | --- | --- | --- |
|  | BMI | Glucose^a^ | Insulin^b^ | HOMA2-IR | CRP | Triglyc.^c^ | HDL Chol.^d^ | LDL Chol.^d^ |
| Glucose^a^ | 0.107* (0.012) |  |  |  |  |  |  |  |
| Insulin^b^ | 0.472*** (<0.001) | 0.325*** (<0.001) |  |  |  |  |  |  |
| HOMA2-IR | 0.465*** (<0.001) | 0.390*** (<0.001) | 0.997*** (<0.001) |  |  |  |  |  |
| CRP | 0.392*** (<0.001) | 0.012 (0.824) | 0.232*** (<0.001) | 0.223***  (<0.001) |  |  |  |  |
| Triglyc.^c^ | 0.246*** (<0.001) | 0.097 (0.062) | 0.373*** (<0.001) | 0.371*** (<0.001) | 0.319*** (<0.001) |  |  |  |
| HDL Chol.^d^ | -0.310*** (< 0.001) | -0.099 (0.073) | -0.261*** (<0.001) | -0.257*** (<0.001) | -0.269*** (<0.001) | -0.273*** (<0.001) |  |  |
| LDL Chol.^d^ | 0.063 (0.297) | 0.041 (0.456) | 0.093 (0.094) | 0.093 (0.094) | 0.136 (0.110) | 0.288*** (<0.001) | -0.162** (0.003) |  |
| Total Chol.^d^ | 0.051 (0.376) | 0.067 (0.195) | 0.147** (0.005) | 0.150** (0.004) | 0.158 (0.055) | 0.477*** (<0.001) | 0.155** (0.005) | 0.872*** (<0.001) |
| **Note.** ***<0.001 **<0.01 *<0.05.  ^a^ Glucose = Fasting Glucose. ^b^ Insulin = Fasting Insulin. ^c^ Triglyc. = Triglycerides. ^d^ Chol. = Cholesterol. | | | | | | | | |

| **Table S3.**  Partial correlations between immune-metabolic markers controlled for age, gender and BMI (*r*(*p*)).   \|  \| Glucose^a^ \| Insulin^b^ \| HOMA2-IR \| CRP \| Triglyc.^c^ \| HDL Chol.^d^ \| LDL Chol.^d^ \| \| --- \| --- \| --- \| --- \| --- \| --- \| --- \| --- \| \| Insulin^b^ \| 0.313***  (<0.001) \|  \|  \|  \|  \|  \|  \| \| HOMA2-IR \| 0.387*** (<0.001) \| 0.996*** (<0.001) \|  \|  \|  \|  \|  \| \| CRP \| -0.033 (0.554) \| 0.058 (0.298) \| 0.050 (0.368) \|  \|  \|  \|  \| \| Triglyc.^c^ \| 0.074 (0.199) \| 0.300*** (<0.001) \| 0.299*** (<0.001) \| 0.249** (0.002) \|  \|  \|  \| \| HDL Chol.^d^ \| -0.069 (0.248) \| -0.137* (0.021) \| -0.134* (0.025) \| -0.168* (0.047) \| -0.214*** (<0.001) \|  \|  \| \| LDL Chol.^d^ \| 0.035 (0.563) \| 0.072 (0.234) \| 0.072 (0.234) \| 0.121 (0.155) \| 0.282*** (<0.001) \| -0.150* (0.012) \|  \| \| Total Chol.^d^ \| 0.062 (0.276) \| 0.140* (0.014) \| 0.143* (0.012) \| 0.150 (0.069) \| 0.480*** (<0.001) \| 0.179** (0.003) \| 0.871*** (<0.001) \| |
| --- | --- | --- | --- | --- | --- | --- | --- | --- | --- | --- | --- | --- | --- | --- | --- | --- | --- | --- | --- | --- | --- | --- | --- | --- | --- | --- | --- | --- | --- | --- | --- | --- | --- | --- | --- | --- | --- | --- | --- | --- | --- | --- | --- | --- | --- | --- | --- | --- | --- | --- | --- | --- | --- | --- | --- | --- | --- | --- | --- | --- | --- | --- | --- | --- |

**Table S4.**

Post-hoc pairwise analysis of significant groups differences in demographic and metabolic variables (I-J) based on estimated marginal means (Bonferroni adjusted).

|  | Hyperarousal anxious-depression low BMI | | | | Hyperarousal anxious-depression high BMI | | |
| --- | --- | --- | --- | --- | --- | --- | --- |
|  | - Hyperarousal anxious-depression high BMI | | - Circadian-bipolar spectrum | | | - Circadian-bipolar spectrum | |
|  | *Estimate* | *Sig.* | *Estimate* | *Sig.* | | *Estimate* | *Sig.* |
| *Age* | -0.68 | 0.09 | -1.84 | <0.001*** | | -1.16 | <0.01** |
| *Gender* | -0.18 | <0.01 | 0.02 | >0.99 | | 0.20 | <0.01** |
| BMI | -1.56 | <0.001*** | -0.75 | <0.001*** | | 0.81 | <0.001*** |
| Fasting Glucose | -0.06 | >0.99 | -0.36 | 0.02* | | -0.30 | 0.13 |
| Fasting Insulin | -0.63 | <0.001*** | -0.40 | <0.01** | | 0.24 | 0.21 |
| HOMA2-IR | -0.45 | <0.001*** | -0.30 | <0.01** | | 0.16 | 0.27 |
| CRP | -0.55 | <0.01** | -0.22 | 0.83 | | 0.34 | 0.33 |
