# Supplementary material for: Identifying mood disorder subgroups at early risk of metabolic dysfunction: a cross-sectional cohort study in young people at early intervention services
Source: BMJ Open. 2025 Sep 26;15(9):e097140. doi: 10.1136/bmjopen-2024-097140 (PMC12481334; doi:10.1136/bmjopen-2024-097140)
Supplement: Supplementary file 3 [file bmjopen-2024-097140.reviewer_comments.pdf]

PEER REVIEW HISTORY

BMJ Open publishes all reviews undertaken for accepted manuscripts. Reviewers are asked to complete a checklist review form and are provided with free text boxes to elaborate on their assessment. These free text comments are reproduced below.

ARTICLE DETAILS

Title (Provisional)

Identifying mood disorder subgroups at early risk of metabolic dysfunction; a cross sectional cohort study in young people at early intervention services.

Authors

McKenna, Sarah; Shin, Mirim; Park, Shin Ho; Nichles, Alissa; Zmicerevska, Natalia; Crouse, Jacob; Janiszewski, Connie; Park, Minji; Phung, Elizabeth; Iorfino, Frank; Varidel, Mathew; Scott, Elizabeth M; Carpenter, Joanne Sarah; Hickie, Ian B

VERSION 1 - REVIEW

**Reviewer**	**1**
**Name**	**Kuula, Liisa**
**Affiliation**	**University of Helsinki Faculty of Medicine**
**Date**	**23-Apr-2025**
**COI**	**None**

The initial sample size is impressive, and allows to truly identify different pathophysiological subtypes as proposed by the authors.

However, this paper seems to contain secondary analyses on an undocumented or vague classification of symptoms, and thus the findings may not be representative.

Several questions emerge regarding the methods and reporting:

1. How divided (across time) was the accumulation of cases?

It seems that the first subgroup is by far the most common - was this true across the 20 years of data collection, or were there time-related trends in subtypes?

2. How is circadian rhythm disruption defined, and does it take into consideration developmental aspects?

3. How is metabolic risk operationalized, and why? There are several markers within the data; the selection should be justified early on.

4. What is the direction of associations - if mood disorders 'emerge' and metabolic risks 'increase', is this something that simply co-occurs, or does one precede the other? The study

setting does not allow using such dynamic directions, and the title should not use the word 'circadian rhythm' if it has not been evaluated and reported systemically.

5. p3, r10-11 Please give a reference that supports 'often attributed to weight gain resulting from medication'

6. p3, r27-28 What is reduced energy in this context?

7. p3, r50-51 Is BMI really the best measure for pCVD risk to compare in these groups? As you state (p4, r 24-25) age-specific BMI explains only 22% of HOMA2-IR; please mention this already on p3, r50-51.

8. p4, r7 Please rephrase 'urgently'

9. p4, r41 Please correct 'he' -> 'we'

10. p5, r47-48 What is the circadian element in the allocation to 'circadian-bipolar' subtype? Would it be possible for the authors to measure a circadian component across all participants, and investigate how having that as a symptom explains metabolic measures?

11. The naming and allocation process overall seems somewhat arbitrary and should be opened up a lot more as it is a key method in this paper. Furthermore, as the data have been collected during 20 years it seems likely that time should also be considered as a potential covariate: research personnel change, digital lifestyle is very different now vs. in 2004, etc. The allocation process may represent a significant bias, and this should be mentioned in the manuscript (and in the STROBE statement).

12. p6, r14 onwards. If the authors have access to all blood works, why focus and hypothesize mainly based on HOMA2? They could easily calculate a risk score or apply some other statistical method that takes into consideration lipids and BMI as well.

13. p6, r55 onwards. The amount of missing data is alarming - why was it so high? Is the reported amount of participants (and statistical power) misleading, or are the missing values truly only missing from one measured variable? How reliable was weight (and height), if some were self-reported and others directly measured?

14. p7, r22 delete extra comma.

15. p7, r45 replace period with comma, and likewise later in the paragraph (r46).

16. Figure 2 - All the participants seem to be relatively high in BMI. How does this compare to previous studies in similar populations?

17. How did the three subtype groups differ regarding gender and age? Differences are presented in supplementary materials, but should be reported in results as there seems to be significant differences in these variables. Perhaps stratification according to gender (or sex - how was data obtained: self-reported or based on some other evaluation?) would have resulted in something revealing about the associations.

18. p11, r45 please correct the direction of >

19. p12, r12-15 please edit syntax

20. p12 Please discuss the clinical significance of the detected differences

21. p12, r30 The reference must be wrong, as 17 is a study on mice (and they cannot have depression as a primary diagnosis).

22. p12, r31 is the explanatory percentage of 22% related to BMI or weight? The paper you refer to is a study on mice and does not seem to be correct for this claim - this should be replaced with a relevant study on humans.

23. p12, r43 The relevance of this paragraph depends on how the authors have operationalized circadian disruption. Based on the text, the operationalization is not particularly accurate and does not contain a continuous component. Thus, bringing up studies that have focused on circadian disruption specifically is not advisable as it may mislead the reader.

24. p13, r7 Did the authors use weight as an individual variable, or was it always BMI?

25. p13, r41-53 no need to repeat numbers

26. p15-16 It seems that references 13 and 26 are actually the same paper, please check all references.

27. The STROBE checklist is somewhat off - there are a few missing reports/ page numbers.

28. Table S4 Correct 'Pos-hoc' -> 'Post-hoc'. Also, as there is some mean differences between the groups, it is likely that th ep-value is not exactly 1, but >.99.

Reviewer 2

Name Dollish, Hannah K

Affiliation Department of Psychiatry, University of Pittsburgh School of Medicine, Psychiatry

Date 16-May-2025

COI None

The study is missing most of the data that was needed to reach statistical power. The authors use pairwise deletion but even that will not accurately reflect the results for 75% of your data missing. The authors also do not explain why this data is missing. The authors do not provide names of the clinics or ethics committees that participated in the study. There is no mention as to what the distribution of the population is (i.e. how many of each gender each group has, what the ethinicity was, the age range within each group,etc.) This is a simple table that is easy to generate and I am concerned as to why the authors omitted it. Lastly they do not address any of the circadian aspects of this paper. They did a singlular blood draw at an unknown time and it may not have even been a consistant time which is essential

for circadian research. There are many issues with their definition of these 3 sub groups and they do not properly address why they are only looking at circadian symptoms in bi-polar participants and not anxiety and neurodevelopmental-psychosis groups. Disorders and conditions within these groups are heavily influced by or have influence over circadian rhythms.

VERSION 1 - AUTHOR RESPONSE

REVIEWER 1:

Dr. Liisa Kuula, University of Helsinki Faculty of Medicine

Comments to the Author:

The initial sample size is impressive, and allows to truly identify different pathophysiological subtypes as proposed by the authors.

However, this paper seems to contain secondary analyses on an undocumented or vague classification of symptoms, and thus the findings may not be representative.

Several questions emerge regarding the methods and reporting:

1. How divided (across time) was the accumulation of cases?

It seems that the first subgroup is by far the most common - was this true across the 20 years of data collection, or were there time-related trends in subtypes?

We agree that it is important to identify any bias that may have arisen from time-related trends. We have provided new information in our supplementary materials including Table S1 describing the proportion of cases who were assigned to each group per year, and Figure S1 showing the number of cases assigned to each group per year.

As shown, whist there are some differences, across time most individuals were assigned to the “hyperarousal anxious-depression” illness group, while the “circadian-bipolar” group is the next more frequent. As noted in the manuscript, individuals are assigned to the “hyperarousal anxious depression” group if they have anxiety and depressive symptoms but no clear manic, atypical features, or psychotic symptoms. The low proportion of individuals assigned to other groups is expected given the low prevalence of these more severe symptoms within the general population, and given we looked at a youth populations who may not yet have developed more severe presentations. Additionally, we have previously shown that 13% of individuals in the anxious-depression subgroup will transition to another

illness group over the course of their care as their illness progresses and they develop more severe symptoms such as mania or psychosis.2

2. How is circadian rhythm disruption defined, and does it take into consideration developmental aspects?

Group assignments are made by a trained clinical researcher, who reviews any available clinician notes, self-report measures and clinical interviews when making group assignments. They are also able to take into account normal developmental changes in sleep-wake functioning. As we have reported in previous publications3-5 the proposed clinical features of mood disorders underpinned by circadian dysfunction include sleep-wake cycle abnormalities (delayed sleep phase, prolonged sleep period and reduced daytime activity, and high variability in daily timing of sleep), low subjective energy and prolonged fatigue not restored by sleep, reduced objective rating of physical activity during daytime, motor agitation, increased appetite or weight gain, and somatic symptoms. While circadian disturbance is the proposed mechanism underlying the circadian-bipolar subgroup, the classification is based on clinical presentation, and so focusses on specific features likely to arise from abnormal circadian functioning i.e. prolonged fatigue, reduced activation, low energy.

Individuals with symptoms of manic-like or atypical depressive symptoms according to existing diagnostic criteria are be assigned to the “circadian-bipolar” group.

We have provided further details in our Methods section regarding how individuals were assigned on page 5:

Assignments are based on a description of the individuals entire clinical history from various data sources including clinician notes, structured clinical interviews, and self-report data, taking into account normative developmental changes.

We have also added further detail about proposed circadian features of mood disorders on page 5:

Any cases with significant manic-like symptoms (manic, hypomanic, or brief hypomanic phenomena) or significant atypical features (consistent with existing diagnostic criteria6,7; e.g., reduced activation, low energy or prolonged fatigue not restored by sleep, increased appetite or weight gain) are allocated to the ‘circadian-bipolar spectrum’ subtype.

3. How is metabolic risk operationalized, and why? There are several markers within the data; the selection should be justified early on.

We thank the reviewer for this comment and agree with the need to make a strong case for preferencing HOMA2-IR as a marker of metabolic risk.

To address this problem, we have made substantial revisions to our Introduction to more clearly show the benefits of monitoring HOMA2-IR as compared to other more common measures such as BMI and fasting glucose:

Page 3:

Current cardiometabolic guidelines for early intervention services emphasise monitoring of 'downstream' markers of metabolic dysfunction, such as BMI, hip/waist circumference, and fasting glucose, rather than early markers such as insulin resistance.8,9 However, young people in in-patient settings with more severe mental illnesses have significantly elevated insulin resistance (measured by HOMA2-IR, a measure of insulin-glucose homeostasis based on fasting insulin and glucose) as compared to those in ambulatory care despite not having raised BMI nor raised fasting glucose.10-12 Only 22% of the variance in HOMA2-IR was accounted for by BMI.10 Thus, although current guidelines emphasise monitoring of BMI and fasting glucose, these may only become elevated later in the course of illness after metabolic dysfunction has persisted for a prolonged period.13,14 Increased monitoring of sensitive early markers of metabolic dysfunction (such as HOMA2-IR) could drastically improve early detection and prevention of physical health comorbidities in youth with major mood disorders.2,10

4. What is the direction of associations - if mood disorders 'emerge' and metabolic risks 'increase', is this something that simply co-occurs, or does one precede the other? The study setting does not allow using such dynamic directions, and the title should not use the word 'circadian rhythm' if it has not been evaluated and reported systemically.

We agree that this study does not address the directionality of the relationship between circadian-bipolar disorders and heightened insulin sensitivity. Further, we did not look at whether circadian disruption is the key driver of poorer metabolic outcomes for the circadian-bipolar group. Again, whilst we are continuing to look at the involvement of circadian disruption in these illnesses and associated comorbidities, we acknowledge further evidence is needed.

To address this problem, we have removed sections of the Introduction that implied that this is what we were testing.

We have also included the following line on page 3:

It is also unknown whether these problems co-occur and are driven by similar mechanisms, or if they are causally related.

Further, the title of our paper has now been changed according to journal guidelines and to better reflect the aims of our paper. We are no longer referencing circadian rhythms.

“Identifying mood disorder subgroups at early risk of metabolic dysfunction; a cross sectional cohort study in young people at early intervention services.”

In the Discussion, we have also described the need for more longitudinal research that can help to establish the causal mechanisms involved in the relationship between mood disorders and poor metabolic outcomes:

Page 13 – page 14:

Another key finding of this study is that young people with circadian-bipolar type illness have heightened risk of metabolic dysfunction as compared to those with hyperarousal-anxious depression. This is consistent with findings from adult populations that rates of metabolic syndrome and pCVD are highest amongst those with bipolar and atypical mood disorders.15,16 Given that this study was cross-sectional, we cannot draw conclusions about which mechanisms are driving the observed differences between the groups. However, ongoing research is focused on the extent to which circadian rhythm disruption is driving “circadian-bipolar spectrum” type mood disorders.3,17 Atypical and bipolar mood disorders are characterized by core clinical features, including hypersomnia, daytime fatigue, non-restorative sleep, reduced motor activity, somatic symptoms, and appetite and weight change that are associated with circadian rhythm disruptions.3,17 Relationships between disrupted circadian systems and metabolic disruption (including insulin resistance) have also been widely reported.18,19 Detailed monitoring and longitudinal research in youth cohorts, tracking trajectories in mood disorder progression and comorbid physical health problems, will lead to improved understanding of potential mediating or moderating mechanisms underlaying this relationship.

5. p3, r10-11 Please give a reference that supports 'often attributed to weight gain resulting from medication'

We apologise for the oversight, this line has been removed during rewrite of the introduction, so citations are no longer necessary.

6. p3, r27-28 What is reduced energy in this context?

The clinical researcher considers, based on clinician reports, self-reports and structured interviews, whether the individual has displayed symptoms of atypical depression consistent with existing diagnostic criteria. ICD and DSM diagnostic criteria for atypical depression including reduced energy or prolonged fatigue.

We have included further clarification on page 5 about the information used to assign individuals to groups:

Assignments are based on a description of the individuals entire clinical history from various data sources including clinician notes, structured clinical interviews, and self-report data, taking into account normative developmental changes.

We have also included the following clarifier on page 5, line 30:

…significant atypical features (consistent with existing diagnostic criteria;

7. p3, r50-51 Is BMI really the best measure for pCVD risk to compare in these groups? As you state (p4, r 24-25) age-specific BMI explains only 22% of HOMA2-IR; please mention this already on p3, r50-51.

We agree with the reviewer that BMI alone is not a sufficiently useful measure of risk for metabolic syndrome and related conditions such as pCVD. Our own research has shown that it only explains a small proportion of the variance in HOMA2-IR.

Despite this, BMI and fasting glucose continue to be the most recommended measure of metabolic health in existing guidelines, including when monitoring young people at early intervention mental health services.13 For this reason, this paper directly compares BMI to other measures of insulin sensitivity, including fasting insulin and HOMA2-IR, to explore whether those who are most at risk of developing metabolic dysfunction show insulin resistance before showing increased weight gain or fasting glucose.

We have added the following qualifier to page 6:

Although we have noted that BMI may not be a sensitive marker of metabolic health, it remains the most commonly used measure of metabolic health in early intervention settings, so was also collected.

8. p4, r7 Please rephrase 'urgently'

This sentence has been replaced as part of our revisions.

9. p4, r41 Please correct 'he' -> 'we'

We apologise for the oversight and have corrected our mistake.

10. p5, r47-48 What is the circadian element in the allocation to 'circadian-bipolar' subtype? Would it be possible for the authors to measure a circadian component across all participants, and investigate how having that as a symptom explains metabolic measures?

We appreciate the question and agree that it is a priority to directly examine how circadian factors are related to metabolic outcomes in individuals with mood disorders, particularly in illness groups with a higher prevalence of metabolic syndrome and pCVD. We have recently published data showing differences in sleep-wake rhythms across all three groups, although more longitudinal research is needed particularly during key illness periods (i.e. manic or psychotic episodes) to better understand how these disturbances are related to illness progression.20 Given that this data was collected over twenty years through various study protocols, we do not have consistent measures of rest-activity patterns or other measures of circadian functioning, as such as we are not able to assess whether this mechanism explains higher levels of HOMA2-IR and BMI that were found in the "circadian-bipolar" group.

To address this problem, we have been clearer in the Introduction sections about the key aims of our paper. The central contribution of our paper is demonstrating that, in a youth population, fasting insulin, glucose and HOMA2-IR levels are elevated in an illness group with atypical and manic symptoms – both of which are associated with a higher prevalence of metabolic syndrome and pCVD in older adult populations.15,16 We have also removed sections of the introduction that implied we were testing whether circadian disruptions are driving metabolic dysfunction in certain groups.

11. The naming and allocation process overall seems somewhat arbitrary and should be opened up a lot more as it is a key method in this paper. Furthermore, as the data have been collected during 20 years it seems likely that time should also be considered as a potential covariate: research personnel change, digital lifestyle is very different now vs. in 2004, etc. The allocation process may represent a significant bias, and this should be mentioned in the manuscript (and in the STROBE statement).

We thank the reviewer for their suggestion and agree it is important to be clearer about how individuals were allocated to groups, we have provided more detail in the Methods section as described above. We have also provided more information in the STROBE statement that the allocation process may represent a significant bias, and we have provided further information in our supplementary materials regarding group allocations per year. Finally, we have made it more clear how criteria for illness group allocations overlap with existing diagnostic criteria.

12. p6, r14 onwards. If the authors have access to all blood works, why focus and hypothesize mainly based on HOMA2? They could easily calculate a risk score or apply some other statistical method that takes into consideration lipids and BMI as well.

As detailed above, we believe that HOMA2-IR is a more sensitive early indicator of metabolic dysfunction, as compared to BMI or fasting glucose that are routinely used to monitor metabolic health. We focus on this marker in order to test this hypothesis and thus to strengthen our case that HOMA2-IR should be routinely monitored in early intervention mental health services.

13. p6, r55 onwards. The amount of missing data is alarming - why was it so high? Is the reported amount of participants (and statistical power) misleading, or are the missing values truly only missing from one measured variable? How reliable was weight (and height), if some were self-reported and others directly measured?

We agree with the reviewers comment that having a large amount of missing data is problematic and have addressed this issue in our Limitations section as well as noting it in the STROBE statement. We have reported on page 4 that the data for this study was collected under two separate ethics protocols. On page 6, lines 6 to 9 we have also provided the following clarification that only participants from our latest study protocol provided full metabolic blood data:

All participants in this study provided fasting plasma glucose (FG) and fasting insulin (FI), A subset of participants from the Neurobiology Youth Follow-up Study also provided cholesterol, triglycerides, and systemic inflammation (CRP).

14. p7, r22 delete extra comma.

Thank you for detecting this, we have removed the comma.

15. p7, r45 replace period with comma, and likewise later in the paragraph (r46).

Thank you, we have made the requested changes on page 6.

16. Figure 2 - All the participants seem to be relatively high in BMI. How does this compare to previous studies in similar populations?

Based on most recent data from the Australian Institute of Health and Wellness, the proportion of individuals in our study who were overweight or obese (BMI ≥ 25) is lower as compared to the general population. Overall, 17.59% of individuals in our study were

overweight or obese, that are lower than rates reported in the AIHW report showing 42.1% of individuals between 18 and 24 have BMI ≥ 25 and 26-29% of 12 to 18 year olds.

17. How did the three subtype groups differ regarding gender and age? Differences are presented in supplementary materials, but should be reported in results as there seems to be significant differences in these variables. Perhaps stratification according to gender (or sex - how was data obtained: self-reported or based on some other evaluation?) would have resulted in something revealing about the associations.

As per the reviewers' suggestion we have now moved our descriptive tables to the main text and summarised gender and age differences in the Results section. We have also described the proportion of individuals who were assigned to each group based on age and gender in Table 1, page 8. As can be seen from this information, it was not possible to stratify analyses as small group sizes would have led to analyses being greatly underpowered.

18. p11, r45 please correct the direction of >

This has been corrected on page 12.

19. p12, r12-15 please edit syntax

20. p12 Please discuss the clinical significance of the detected differences

We have revised this paragraph, partly to discuss the clinical significance of the differences we detected in insulin resistance. We hope this has resolved both issues.

21. p12, r30 The reference must be wrong, as 17 is a study on mice (and they cannot have depression as a primary diagnosis).

22. p12, r31 is the explanatory percentage of 22% related to BMI or weight? The paper you refer to is a study on mice and does not seem to be correct for this claim - this should be replaced with a relevant study on humans.

All references have been checked. The references in question have been replaced due to revisions to the text.

23. p12, r43 The relevance of this paragraph depends on how the authors have operationalized circadian disruption. Based on the text, the operationalization is not particularly accurate and does not contain a continuous component. Thus, bringing up studies that have focused on circadian disruption specifically is not advisable as it may mislead the reader.

We agree with the reviewers criticism and as shown above, have revised the paragraph (page 13 – page 14). As discussed previously, we did not assess circadian disruption in this paper and thus it is potentially misleading to suggest that this mechanism is the reason why our “circadian-bipolar” illness group had elevated insulin resistance.

24. p13, r7 Did the authors use weight as an individual variable, or was it always BMI?

We have only used BMI within this study, we have provided further clarification in our Methods on page 6:

(weight alone was not included in our analyses)

25. p13, r41-53 no need to repeat numbers

We have revised these sentences so there is no need to repeat reference numbers.

26. p15-16 It seems that references 13 and 26 are actually the same paper, please check all references.

We apologise again for the oversight and have updated our references.

27. The STROBE checklist is somewhat off - there are a few missing reports/ page numbers.

Thank you to the reviewer for bringing this to our attention and we apologise for the oversight. We have now revised the STROBE checklist.

28. Table S4 Correct 'Pos-hoc' -> 'Post-hoc'. Also, as there is some mean differences between the groups, it is likely that th ep-value is not exactly 1, but >.99.

Thank for you detecting this, we have made the requested change.

REVIEWER 2

Dr. Hannah K Dollish, Department of Psychiatry, University of Pittsburgh School of Medicine

Comments to the Author:

The study is missing most of the data that was needed to reach statistical power.

Our power analysis showed that we needed at least 100 participants in each group to detect significant differences, one group far exceeded this number (hyperarousal-anxious depression, n = 656), and one group was only slightly lower (circadian-bipolar, n = 95). However, we agree that the neurodevelopmental-psychosis group (n = 39) was significantly smaller than required. Our analysis was therefore underpowered to detect differences between the neurodevelopmental-psychosis group and other illness groups, suggesting it is important we go back and look at this particular group in more detail in the future.

Despite this limitation, our study has been able to identify that the circadian-bipolar illness subtype is associated with increased insulin resistance even in young adults. As such, it is important to monitor at risk subgroups in early intervention services. With further research, this may include the neurodevelopmental-psychosis subgroup.

To address this issue, we have noted that our analyses was underpowered in the Limitations section page 15:

Certain analysis had greatly increased statistical power as compared to others. More importantly, based on our power analyses, the neurodevelopmental-psychosis group was underpowered to detect a moderate effect.

The authors use pairwise deletion but even that will not accurately reflect the results for 75% of your data missing. The authors also do not explain why this data is missing.

We agree with the reviewer that our analysis was limited by the high rates of missing data for certain variables. As mentioned above, we have provided further clarification in the text that some of our study protocols did not involve collecting blood lipids (e.g. cholesterol and triglycerides). Our most recent and ongoing study protocols have included these measures which should allow us to conduct more detailed analyses in future research.

On page 6 we have provided the following clarification:

All participants in this study provided fasting plasma glucose (FG) and fasting insulin (FI), A subset of participants from the Neurobiology Youth Follow-up Study also provided cholesterol, triglycerides, and systemic inflammation (CRP).

The authors do not provide names of the clinics or ethics committees that participated in the study.

We apologise for the oversight and have now included identifying information in the manuscript, see page 4.:

Ethical approval for the study was obtained from the Human Research Ethics Committee of the Sydney Local Health District (2020/ETH01272). Informed consent was secured from all

participants. A subset of participants from the Neurobiology Youth Follow-Up Study also consented to share their historical blood data from a prior study approved by the Human Research Ethics Committee of the University of Sydney (2012/1631). The current paper is reported according to STROBE guidelines (see supplementary materials).1

There is no mention as to what the distribution of the population is (i.e. how many of each gender each group has, what the ethinicity was, the age range within each group,etc.) This is a simple table that is easy to generate and I am concerned as to why the authors omitted it.

Some of this information was previous provided in Supplementary tables however has now been moved to the main text as per suggestions from both reviewers. Unfortunately, we do not have information on participants ethnicity, we have also included the following information on page 14 of our discussion:

Important confounders, most notably medication use or concurrent medical conditions, and sociodemographic variables such as ethinicity, were not accounted for in the analysis.

Lastly they do not address any of the circadian aspects of this paper. They did a singular blood draw at an unknown time and it may not have even been a consistant time which is essential for circadian research. There are many issues with their definition of these 3 sub groups and they do not properly address why they are only looking at circadian symptoms in bi-polar participants and not anxiety and neurodevelopmental-psychosis groups. Disorders and conditions within these groups are heavily influenced by or have influence over circadian rhythms.

As noted above, we agree with the reviewers' criticism. We have revised our manuscript so that it is more clear to readers that we did not test the underlaying mechanisms that may explain why the Circadian-Bipolar group had high insulin resistance. We have also provided more detail about how we assigned individuals into each illness group, and made it more clear what circadian symptoms were considered. We have revised the paragraph on page 13, lines 29 to 34 and page 14, lines 1 to 9, and have provided the new paragraph above.

Thank you again for the review and the opportunity to resubmit. We have attempted to address all of your editorial and reviewers' comments and have noted when and where we have made changes in our revised manuscript. We have found these thoughtful comments most helpful and we hope you agree that the paper is now acceptable for publication.

Kind regards,

Sarah McKenna

REFERENCES

1. von Elm E, Altman DG, Egger M, Pocock SJ, Gøtzsche PC, Vandenbroucke JP. The Strengthening the Reporting of Observational Studies in Epidemiology (STROBE) statement: guidelines for reporting observational studies. Ann Intern Med. Oct 16 2007;147(8):573-7. doi:10.7326/0003-4819-147-8-200710160-00010

2. Hickie IB, Scott EM, Cross SP, et al. Right care, first time: a highly personalised and measurement-based care model to manage youth mental health. Medical Journal of Australia. 2019;211(S9)doi:10.5694/mja2.50383

3. Crouse JJ, Carpenter JS, Song YJC, et al. Circadian rhythm sleep–wake disturbances and depression in young people: implications for prevention and early intervention. The Lancet Psychiatry. 2021;8(9):813-823. doi:10.1016/s2215-0366(21)00034-1

4. Hickie IB, Hermens DF, Naismith SL, et al. Evaluating differential developmental trajectories to adolescent-onset mood and psychotic disorders. BMC Psychiatry. 2013/11/12 2013;13(1):303. doi:10.1186/1471-244X-13-303

5. Hickie IB, Naismith SL, Robillard R, Scott EM, Hermens DF. Manipulating the sleep-wake cycle and circadian rhythms to improve clinical management of major depression. BMC Medicine. 2013/03/22 2013;11(1):79. doi:10.1186/1741-7015-11-79

6. Association AP, ed. Diagnostic and statistical manual of mental disorders (5th ed., text rev.). 2022.

7. Organisation” WH. International statistical classification of diseases and related health problems (11th ed.). 2019.

8. McIntyre RS, Kwan ATH, Rosenblat JD, Teopiz KM, Mansur RB. Psychotropic Drug–Related Weight Gain and Its Treatment. American Journal of Psychiatry. 2024/01/01 2024;181(1):26-38. doi:10.1176/appi.ajp.20230922

9. Alonso-Pedrero L, Bes-Rastrollo M, Marti A. Effects of antidepressant and antipsychotic use on weight gain: A systematic review. Obesity Reviews. 2019/12/01 2019;20(12):1680-1690. doi:https://doi.org/10.1111/obr.12934

10. Scott EM, Carpenter JS, Iorfino F, et al. What is the prevalence, and what are the clinical correlates, of insulin resistance in young people presenting for mental health care? A cross-sectional study. BMJ Open. 2019;9(5):e025674. doi:10.1136/bmjopen-2018-025674

11. Wilson CE, Carpenter JS, Crouse JJ, et al. Cross-sectional and longitudinal associations between cardiometabolic measures and clinical stage in young people accessing early intervention mental health services. Early Intervention in Psychiatry. 2023;17(9):893-900. doi:10.1111/eip.13381

12. Tickell AM, Rohleder C, Ho N, et al. Identifying pathways to early-onset metabolic dysfunction, insulin resistance and inflammation in young adult inpatients with emerging affective and major mood disorders. Early Intervention in Psychiatry. 2022;16(10):1121-1129. doi:10.1111/eip.13260

13. Curtis J, Newall HD, Samaras K. The heart of the matter: cardiometabolic care in youth with psychosis. Early Intervention in Psychiatry. 2012;6(3):347-353. doi:10.1111/j.1751-7893.2011.00315.x

14. Firth J, Siddiqi N, Koyanagi A, et al. The Lancet Psychiatry Commission: a blueprint for protecting physical health in people with mental illness. The Lancet Psychiatry. 2019;6(8):675-712. doi:10.1016/s2215-0366(19)30132-4

15. Plana-Ripoll O, Weye N, Momen NC, et al. Changes Over Time in the Differential Mortality Gap in Individuals With Mental Disorders. JAMA Psychiatry. 2020;77(6):648. doi:10.1001/jamapsychiatry.2020.0334

16. Goldstein BI, Carnethon MR, Matthews KA, et al. Major Depressive Disorder and Bipolar Disorder Predispose Youth to Accelerated Atherosclerosis and Early Cardiovascular Disease. Circulation. 2015/09/08 2015;132(10):965-986. doi:10.1161/CIR.0000000000000229

17. Carpenter JS, Crouse JJ, Scott EM, et al. Circadian depression: A mood disorder phenotype. Neuroscience & Biobehavioral Reviews. 2021;126:79-101. doi:10.1016/j.neubiorev.2021.02.045

18. Stenvers DJ, Scheer F, Schrauwen P, la Fleur SE, Kalsbeek A. Circadian clocks and insulin resistance. Nat Rev Endocrinol. Feb 2019;15(2):75-89. doi:10.1038/s41574-018-0122-1

19. Zimmet P, Alberti K, Stern N, et al. The Circadian Syndrome: is the Metabolic Syndrome and much more! J Intern Med. Aug 2019;286(2):181-191. doi:10.1111/joim.12924

20. Carpenter JS, Crouse JJ, Park SH, et al. Actigraphy-derived circadian rhythms, sleep-wake patterns, and physical activity across clinical stages and pathophysiological subgroups in young people presenting for mental health care. Journal of Psychiatric Research. 2025/06/01/ 2025;186:396-406. doi:https://doi.org/10.1016/j.jpsychires.2025.03.003

VERSION 2 - REVIEW

Reviewer 2

Name Dollish, Hannah K

Affiliation Department of Psychiatry, University of Pittsburgh School of Medicine, Psychiatry

Date 25-Jun-2025

COI

The authors acknowledge the limitations in their paper and conceed that future studies are needed to answer the question this paper is trying to ask. I do not believe anything with regard to the neurodevelopmental-psychosis group is valid in this paper as the authors acknowledge their group is underpowered for poper statistical analysis. I will conceed on the other groups that no study is perfect and there are many limitations with human research. But I do not believe that data from an underpowered population has any business being included in the main conclusions in the paper. If anything this population should be mentioned in the discussion as "we observed this but did not include" and that is all.

VERSION 2 - AUTHOR RESPONSE

Reviewer: 2

I do not believe anything with regard to the neurodevelopmental-psychosis group is valid in this paper as the authors acknowledge their group is underpowered for proper statistical analysis. I will concede on the other groups that no study is perfect and there are many limitations with human research. But I do not believe that data from an underpowered population has any business being included in the main conclusions in the paper. If anything this population should be mentioned in the discussion as "we observed this but did not include" and that is all.

We have addressed the reviewers concerns by removing the neuro-developmental group from our analyses and providing updated results from new analyses that only compared the hyperarousal-anxious depression and circadian-bipolar spectrum illness groups. We have made changes throughout the paper to reflect this and included two revised Figures.

For transparency, and to ensure that readers have a full understanding of our model, we have defined the neurodevelopmental illness group and described how individuals are assigned to this group in the Introduction and Methods. However, we have also noted in the Results section (page 7) that this group was underpowered and was not included in our analyses. We have also discussed this in our limitations section (page 11).

VERSION 3 - REVIEW

Reviewer 2

Name Dollish, Hannah K

Affiliation Department of Psychiatry, University of Pittsburgh School of Medicine, Psychiatry

Date 12-Aug-2025

COI

None
